# Trends and Spatial Patterns of Oral Cancer Mortality in Ecuador, 2001–2016

**DOI:** 10.1155/2018/6086595

**Published:** 2018-07-02

**Authors:** Solange Núñez-González, J. Andrés Delgado-Ron, Christopher Gault, Daniel Simancas-Racines

**Affiliations:** Centro de Investigación en Salud Pública y Epidemiología Clínica (CISPEC), Universidad Tecnológica Equinoccial, Quito 170129, Ecuador

## Abstract

The aims of this study were to describe the temporal trend of OC from 2001 to 2016 and to analyze the space and space-time clusters of high mortality due to OC in Ecuador from 2011 to 2016. *Methods*. The present study is a mixed ecological study; the time trends were obtained using a Joinpoint regression model, space-time scan statistics was used to identify high-risk clusters, and Global Moran I index was calculated. *Results*. In Ecuador, between 2001 and 2016, OC caused a total of 1,025 deaths. Crude mortality rates significantly increased, with an APC (annual percentage change) of 2.7% (*p*=0.009). The age-standardized mortality rate did not significantly increase (APC: 1.73%; *p*=0.08). The most likely cluster was detected in 2015, included 20 cantons. The second cluster included 38 cantons, in the years 2014 to 2016. The Global Moran I index for the study period showed a negative spatial autocorrelation (−0.067; *p*=0.37). *Conclusion*. Mortality due to OC in Ecuador significantly increased over the 16-year study period, the young groups being the most affected. Ecuadorian provinces present high variability in types of OC and cancer rates.

## 1. Introduction

Oral cancer (OC) is a relevant problem of global public health due to its growing tendency and impact on the young population in the last decades [1, 2]. In 2012, OC and pharyngeal cancer were estimated to be responsible for 529,500 incident cases—3.8% of all cancer cases, and is predicted to rise 62% to 856,000 cases by 2035 [1]. Globally, deaths from OC increased by 2.6% between 2006 and 2016, while age-standardized death rates from OC showed a slight increase of 0.7% within the same period [3].

In South America, 15,868 new cases of lip and oral cavity cancer occurred in 2012, and 6,046 deaths were registered in the same year [4]. Mortality ranged from 0.72 to 6.04 per 100,000 population from 1999 to 2012; it is significantly decreasing in males from 2.5% to 2.1% in Argentina, Chile, Colombia, and Ecuador, while it is significantly increasing in women in Brazil and Peru [5]. Worldwide, the South American region also presented the second lowest mortality rate (1.2 : 100,000) [6].

Risk factors for developing OC include tobacco consumption, either smoking or chewing, and alcohol consumption. Both of these factors account for nearly 90% of the cases and are associated with age, sex, and religion-ethnicity [7]. Recent studies [8–11] suggest that human papilloma virus (HPV) acts as an independent risk factor for OC E6 and E7 oncogenes' mutagenesis, which affects the carcinogenic progression of HPV; the former disrupts the p53 tumor suppression pathway [12], and the latter targets the tumor suppressor retinoblastoma protein [13]. However, the correlation between HPV and OC is inconclusive mainly due to different subsites of the oral cavity being combined as a single entity in national reports [14, 15]. Finally, two independent meta-analyses correlated OC with processed meat consumption (RR  =  1.91, 95% CI (1.19–3.06)) and periodontal disease (OR = 3.21, 95% CI (2.25–4.16)) [16, 17].

The epidemiology of oral cancer is significantly different in men and women. Oropharyngeal cancers caused by HPV are more likely to occur in men without pernicious habits and good socioeconomic status, whereas tongue cancer affects mostly young Caucasian women [2]. The risk of mortality after developing OC is higher in female smokers than in male smokers [18, 19].

It is not clear how much OC impacts Ecuador—most recent data from the National Tumor Registry dates from 2012 and does not include all cities in the country. The National Institute of Statistics and Census (INEC), on the other hand, issues an annual report of mortality classified according to the International Statistical Classification of Diseases and Related Health Problems (nearly a hundred of which attributed to OC in 2016, most of them in the tongue and the parotid glands) [20].

Previous studies have used partial registers when evaluating OC epidemiology in the country; most of them are also outdated [5, 6]. In countries with insufficient data, a conscious analysis is required in order to understand the true impact of the disease, and it is also important to explore patterns of distribution to determine environmental risk factors. No published study analyzes the trend and geographic distribution of OC in Ecuador.

The present study aims at describing the temporal trend of oral cancer from 2001 to 2016 and at analyzing the space and space-time conglomerates of high mortality due to OC in Ecuador from 2011 to 2016.

## 2. Materials and Methods

### 2.1. Data Sources

Data were collected from the INEC database in its “Statistical Reports” section: live birth, death, and fetal death [20]. Death registries include “basic cause” of death coded according to the International Classification of Diseases (ICD), which we used for our temporal analysis. Registries matched ICD-10 codes C00-C10 for OC. Finally, we extracted population data from estimates of 2001 and 2010 censuses conducted by the INEC [21].

### 2.2. Study Area

Ecuador is one of the smaller countries in South America, located on its west coast, and it has a total area of 283,561 km^2^ [22]. It is geographically divided into four regions (the Amazon, the Highlands, the Coast, and the Galapagos Islands) and is politically split into 24 provinces, which, in turn, are split into 224 cantons [23].

### 2.3. Statistical Analysis

Crude mortality rates and specific mortality rates by sex and age group (30–39, 40–49, 50–59, 60–69, 70–79, and ≥80 years) were calculated. We calculated age-standardized mortality rates (ASMR) stratified by sex using the direct standardization method, through the population standard of the World Health Organization (WHO) [24]. All rates are expressed as death per 100,000 population. Microsoft Excel 2010 (Microsoft Office Professional Plus 2010) was used to calculate mortality rates and standard errors. For the time trend analysis, we used the Joinpoint regression model to identify the years when there were significant changes in ASMR and crude mortality rates. Joinpoint regression analysis fits a series of joined straight lines on a logarithmic scale; straight line segments are joined at “Joinpoints,” where mortality trend changes with statistical significance [25]. The slope of each line segment, of the best-fitting model, was expressed as the annual percentage change (APC) and average annual percent change (AAPC). Significance tests were performed using the Monte Carlo permutation technique. We considered *p* statistically significant when below 0.05. Our team used the Joinpoint Regression Program version 4.4.0.0, from the Surveillance Research Program of the US National Cancer Institute, for the statistical analysis [25].

We identified high-risk spatiotemporal clusters of OC mortality in Ecuador during the period 2011–2016 using retrospective Kulldorff's spatiotemporal scan statistics. Under the statistical assumption that mortality cases follow a Poisson distribution, we used a discrete model. The 224 cantons of Ecuador were our spatial unit of analysis, with a maximum spatial cluster size of 30% of the population at risk and a maximum temporal cluster size of 50% of the study period. The most likely or primary cluster and secondary clusters were detected through the log-likelihood ratio (LLR) test [26]. The statistical significance of these clusters was calculated through Monte Carlo simulations. We analyzed spatiotemporal trends using SaTScan software [27] and displayed them in cartographic representations created by free software QGIS 2.18.14. Finally, using Global Moran's I index, we assessed the presence of global spatial autocorrelation. We calculated the average annual crude mortality rates by cantons for the period 2011–2016, to both correct for random fluctuations and provide greater stability of mortality rates in small cantons. We calculated smoothed mortality rates by applying the Local Empirical Bayesian smoothing method [28]. We used Local Index of Spatial Association (LISA) by means of Local Moran's I index to evaluate the existence of local autocorrelation, thus identifying significant hot spots (high values next to high, HH), cold spots (low values next to low, LL), and spatial outliers (high amongst low, HL or vice versa, LH) of mortality rates [29]. For spatial representation of the Local Moran's index, we created Moran maps that include cantons with significant differences. GeoDa software was used for the spatial analysis and smoothed mortality rates calculation (GeoDa Center for Geospatial Analysis and Computation, Arizona State University, Tempe, AZ, USA). Additional cartographic representations were created to showcase this analysis.

## 3. Results

During 2001–2016, 1,025 people died from OC in Ecuador, 52.4% were males and 47.6% females (sex ratio: 1 : 1.1). Reported cases show a mean age of 67 years (±15.2) in men and 70.1 years (±16.2) in women. According to the area of residence, 81.56% (*n*=836) of deaths occurred in urban areas and 18.44% (*n*=189) in rural areas.

**Figure 1:**
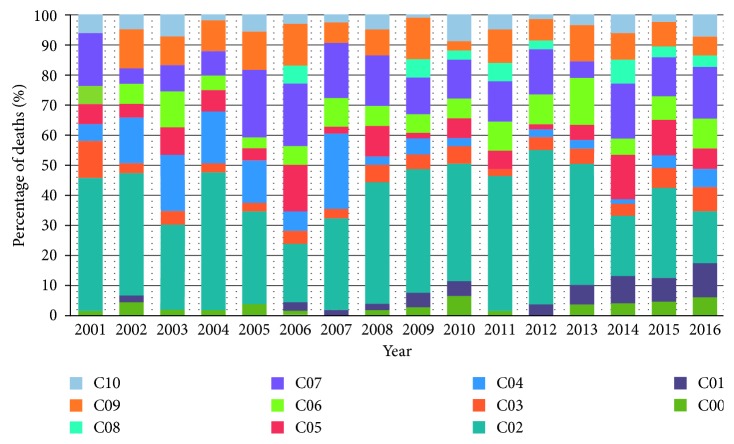
Percentage of deaths due to oral cancer according to the ICD-10 classification per year in Ecuador, 2001 to 2016.

Malignant neoplasm of other and unspecified parts of the tongue represented 34% of deaths (*n*=349), parotid gland tumors 14.8% (*n*=152), and neoplasms of other and unspecified major salivary glands 8.7% (*n*=89). These three ICD-10 codes (C02, C07, and C08) accounted for 57.6% of deaths. We illustrated the annual distribution of deaths by ICD-10 code in (Figure 1).

**Figure 2:**
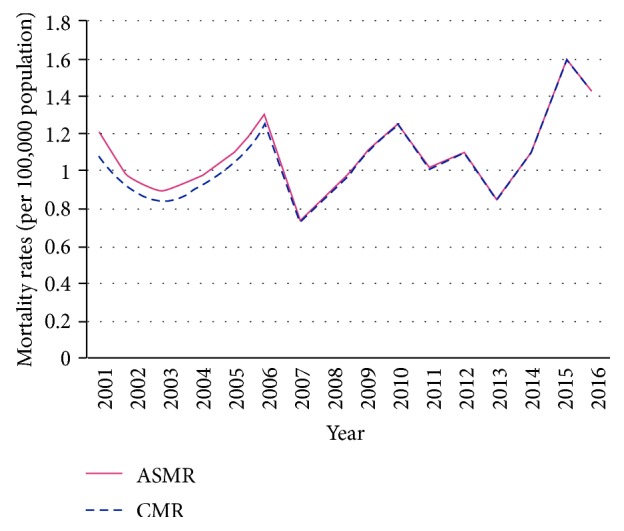
Trends of the crude mortality rates (CMRs) and the age-standardized mortality rate (ASMR) per 100,000 population for oral cancer in Ecuador, 2001 to 2016.

Mortality, in absolute numbers, increased at national level from 50 deaths in 2001 to 103 in 2016. Between these years, crude mortality rates increased from 1.0 to 1.4 per 100,000 population, presenting a significant increasing trend at national level (APC: 2.7%; *p*=0.009) (AAPC: 2.7%; 95% CI: 0.8 to 4.7). ASMR presented a nonsignificant increase from 1.2 to 1.4 per 100,000 population (APC: 1.73%; *p*=0.08) (AAPC: 1.7%; 95% CI: −0.3 to 3.8) over the 16-year study period (Figure 2).

Regression analysis of the specific mortality rates by age group for the period 2001–2016 revealed a significant increase in the APC in younger groups: 30–39 years (5.16%; *p*=0.03) and 40–49 years (4.91%; *p*=0.04). Trends in age groups 50–59 years (1.91%), 60–69 years (0.03%), and 70–79 years (0.88%) were not significant. Finally, the 80–89 years group reported an initial period (2001–2009) of no significant decrease (−5.52%; *p*=0.13), followed by another period (2009–2016) of significant increase (10.29%; *p*=0.01).

Crude mortality rates in male and female increased between 2001 and 2016 from 1.0 to 1.7 and from 1.1 to 1.2 per 100,000 population, respectively. ASMR among men increased from 1.2 in 2001 to 1.7 per 100,000 population in 2016, while ASMR in women barely changed from 1.18 to 1.17 per 100,000 population, in the same period. The Joinpoint analysis for crude mortality and ASMR can be seen in Table 1.

We identified two statistically significant clusters for high occurrence of OC deaths in our 2011–2016 spatiotemporal analysis. The most likely (or “primary”) cluster occurs in 2015; it includes 20 cantons located in 5 provinces (Cotopaxi, Los Ríos, Pichincha, Santo Domingo de los Tsáchilas, and Tungurahua) totaling 56 deaths. Relative risk (RR) was 2.58 (LLR: 17.58, *p* < 0.001), with an annual mortality rate of 1.2 per 100,000 population.

**Figure 3:**
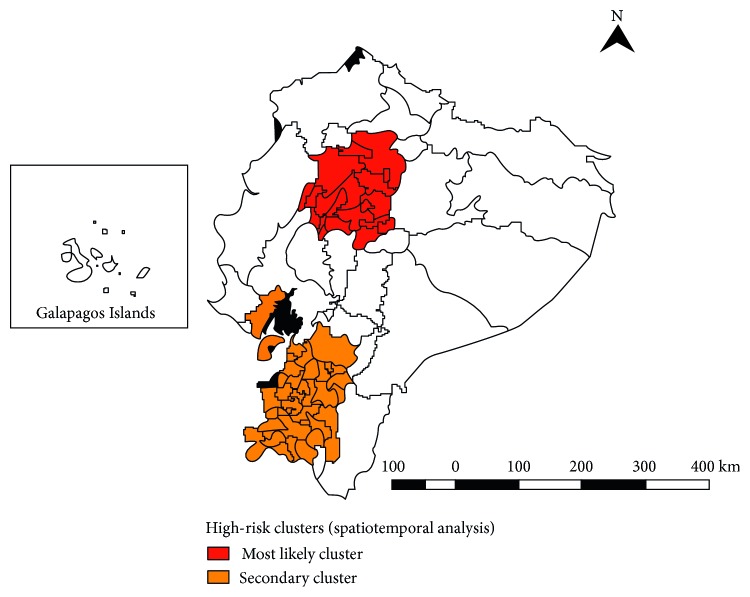
Spatiotemporal cluster analysis of oral cancer mortality rates by cantons in Ecuador, 2000 to 2011.

The secondary high-risk cluster was detected in the years 2014 to 2016 and includes 38 cantons in 4 provinces (Azuay, El Oro, Guayas, and Loja); 106 people died from OC in this cluster. RR was 1.69 (LLR: 10.39; *p*=0.03), and the annual mortality rate was 0.8 per 100,000 population (Figure 3).

**Figure 4:**
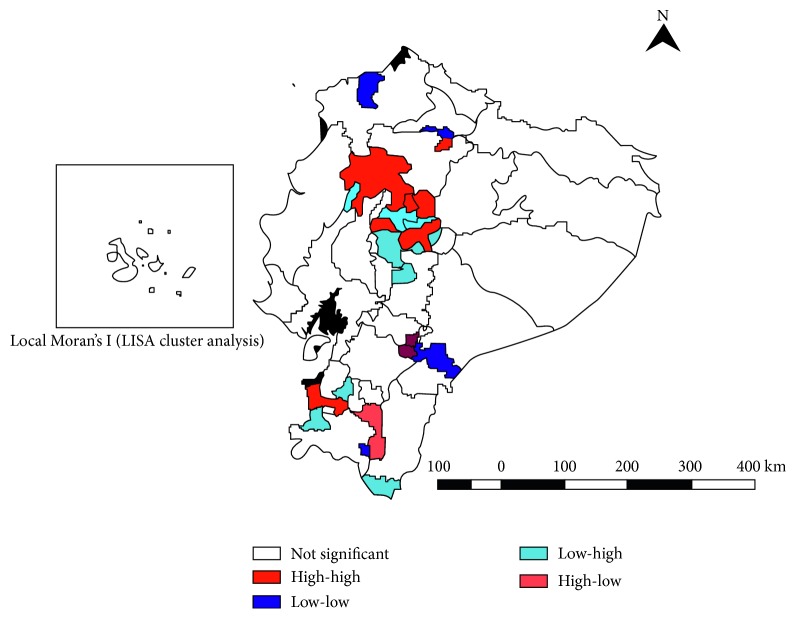
Local Index of Spatial Association (LISA) cluster analysis of oral cancer mortality rates by cantons in Ecuador, 2000 to 2011.

Average annual crude cantonal mortality rates ranged from 0.0 to 3.16 deaths per 100,000 population, and smoothed mortality rates ranged from <0.001 to 3.15 deaths per 100,000 population. The Global Moran I index for the study period shows a negative spatial autocorrelation, although not significant (−0.067; *p*=0.37). We identified a high-risk cluster (High/High) for OC mortality, which includes 12 cantons in 6 provinces (Cotopaxi, Los Ríos, El Oro, Manabí, Santo Domingo de los Tsáchilas, and Tungurahua). Clusters with low rates (Low/Low) include 7 cantons in 5 provinces (Esmeraldas, Imbabura, Azuay, Loja, and Morona Santiago) (Figure 4).

## 4. Discussion

During the study period, mortality rates for OC increased, in both men and women. Our age-group analysis shows a statistically significant increase in mortality in the 30–39 and 40–49 years groups; no significant changes were reported in the 50–59, 60–69, and 70–79 years groups. On the other hand, the ≥80 years group showed an initial decrease followed by a statistically significant period of increased mortality.

We identified spatial and spatiotemporal high-risk clusters for mortality. The primary cluster was mainly located (15 out of 19 cantons) in the Highlands region: Cotopaxi, Pichincha, Santo Domingo de los Tsáchilas, and Tungurahua provinces; the remaining 4 cantons are located in the Coastal province Los Ríos. The secondary high mortality cluster contained 38 cantons, 22 belong to the Highlands region in Azuay and Loja provinces, and 16 cantons belong to the Coast region, in Guayas and El Oro provinces. The highest concentration of deaths was reported in urban areas, similarly to a previous report [30].

Studies in the region [5, 31, 32] show a decrease in mortality trends across countries, Ecuador included. However, the lack of reliable and current databases might cause an underestimation of OC impact. Our findings compared to those of Hussein et al. [2] show a rise of OC in younger populations worldwide; they suggest a different etiology in these patients (tongue cancer affects mainly young white women and oropharyngeal cancer affects men with good socioeconomic status and without pernicious habits). However, our study did not identify differences among men and women related to the location of the tumor. While primary and secondary high-risk clusters identified in our analysis locate in areas with moderate to high levels of consumption (defined as average monthly spending in dollars per inhabitant), more studies are required to determine whether or not socioeconomic factors influence OC mortality in Ecuadorians.

Early detection and screening reduce mortality and morbidity of most common cancers [34, 35]. Screening programs for OC have not been promoted in health services due to the limited available evidence on its effectiveness. However, a systematic review by Brocklehurst et al. reported that a visual exam screening reduces OC mortality rate in high-risk individuals [36]. More studies are required to prove the effectiveness of such intervention.

Late diagnosis—present in at least 50% of cases—worsens patient prognosis and associates with greater mortality [37]. Thus, it is imperative to propose strategies focused on early diagnosis and opportune treatment. The Ecuadorian “National Strategy for Comprehensive Cancer Care” aligns with the goals of the “Action Plan for the Prevention and Control of Noncommunicable Diseases (NCDS) 2013–2020” from OPS and WHO [35, 38]; it was implemented in 2017 to reduce cancer mortality by 25% in the country by 2025. But this strategy lacks a specific focus on oral cancer, which might be added under its strategic line to “organize and implement a timely response for screening, detection, specialized diagnosis to improve the prognosis and life of cancer patients.” Additional lines of action in the strategy-targeting alcohol and tobacco consumption might reduce their effects as risk factors [38]. Similarly, widespread vaccination against HPV might decrease the incidence of OC associated with the virus. We encourage policymakers to adequately evaluate the impact of the interventions mentioned above by age group since “oral cancer” encompasses multiple oncological entities.

The University of Washington's Institute for Health Metrics and Evaluation (IHME) qualified the data quality of INEC's registry with four out of five stars, as reported in the Global Burden of Disease 2016 Study [3].

Use of the Jointpoint regression model for the description of OC mortality trends strengthens this study; this analysis allows us to detect statistically significant changes from 2001 to 2016, which avoids the prespecification of periods by the researcher. Kulldorf's spatiotemporal analysis, on the other hand, identifies the distribution of the disease in time and space, evaluating the statistical significance of clusters in high-risk, low-risk, and all-risk categories; it also detects epidemics early [39].

Limitations of our study lie in the difficulty to associate the observed trends with clinical factors, as well as to associate clusters with factors related to each geographical area. Underreporting is also possible due to potential underdiagnosis of OC as cause of death in the country.

## 5. Conclusion

Mortality due to OC in Ecuador significantly increased over the 16-year study period, contrasting with other countries that show a decrease in mortality crude rates. Ecuadorian provinces present high variability in types of OC and cancer rates. The spatial analysis indicates the presence of high occurrence clusters throughout six provinces of the country: Cotopaxi, Los Ríos, El Oro, Manabí, Santo Domingo de los Tsáchilas, and Tungurahua. Factors associated with this geographic pattern should be studied in order to create and implement policies oriented to decrease OC mortality in Ecuador. Better cancer registers should be developed to improve vigilance of oncologic diseases in the country.

## Figures and Tables

**Table 1:** Joinpoint analysis of the crude mortality rates and the age-standardized oral cancer mortality rates by sex in Ecuador, 2001 to 2016.

Variable	0 Joinpoints		1 Joinpoint	
	Year	APC (95% CI)	Year	APC (95% CI)
*CRM*				
Male	2001–2016	2.86^*∗*^ (0.6; 5.2)		
Female	2001–2016	2.55^*∗*^ (0.3; 4.9)	2001–2003	−12.31 (−54.0; 67.1)
			2003–2016	3.66^*∗*^ (0.5; 6.9)

*ASMR*				
Male	2001–2016	1.7 (−0.6; 4.0)		
Female	2001–2016	1.87 (−0.5; 4.3)		

CMR, crude mortality rates; ASMR, age-standardized mortality rates; APC, annual percent change; CI, confidence interval. ^*∗*^The annual percent change is significantly different from 0 (two-sided *p* < 0.05).

## Data Availability

Data for this research were obtained from the National Institute of Statistics and Census of Ecuador. All databases of general and fetal deaths are available online at http://www.ecuadorencifras.gob.ec/nacimientos-defunciones/.
